# Pain intensity and salivary α‐amylase activity in patients following mandibular third molar surgery

**DOI:** 10.1002/cre2.628

**Published:** 2022-07-07

**Authors:** Wanvipa Surin, Piyanart Chatiketu, Nuntouchaporn Hutachok, Somdet Srichairatanakool, Vuttinun Chatupos

**Affiliations:** ^1^ Department of Oral and Maxillofacial Surgery, Faculty of Dentistry Chiang Mai University Chiang Mai Thailand; ^2^ Department of Family and Community Dentistry, Faculty of Dentistry Chiang Mai University Chiang Mai Thailand; ^3^ Department of Biochemistry, Faculty of Medicine Chiang Mai University Chiang Mai Thailand

**Keywords:** α‐amylase, molar surgery, pain, saliva

## Abstract

**Objective:**

We aimed to compare the levels of pain and salivary α‐amylase (SAA) in patients before and after mandibular third molar surgery.

**Materials and Methods:**

Patients were divided into asymptomatic and symptomatic groups and were then identified by the analgesic drug taken throughout the 2‐week study. The visual analog scale (VAS) was employed to evaluate the severity of pain experienced by a given subject before treatment, when the anesthetic wore off, in the morning, and at night for a period of 1 week. Saliva was collected from the mouth floor of the subjects and the levels of SAA activity were measured at indicated times.

**Results:**

The levels of postoperative pain were higher than those of pretreatment pain (*p* < 0.05), but were not necessarily different between the two groups. The pain levels were positively correlated with SAA activities in both groups (*p* < 0.05). There was no difference between the number of analgesics taken by the two groups and the postoperative complications observed during the study. A significant correlation was observed between the VAS pain scale and SAA activities.

**Conclusion:**

SAA would be a simple effective biomarker for the objective assessment of pain intensity in patients who have undergone mandibular third molar surgery.

**Abbreviations**
SAA: salivary α‐amylase
SD: standard deviation
VAS: visual analog scale

## 1 INTRODUCTION

Acute pain experienced as a result of symptomatic wisdom teeth has been identified as a significant problem in the field of maxillofacial surgery. This type of pain is complicated and can be difficult to treat as patients experience extreme discomfort. There are many options for the management of pain in patients with symptomatic wisdom teeth. These options include supportive types of treatments that include antibiotic treatment, the removal of opposing teeth, irrigation around the affected tooth, curettage, excision of the operculum, or the surgical removal of the third molar to remove the source of the infection (Frew, 1937). Leaving the infected tooth in place may allow pathogenic microorganisms to spread into the deep fascia, consequently resulting in airway obstruction and a potentially life‐threatening condition (Kunkel et al., 2004, 2007). Previous studies have reported that severe complications can occur from an immediate extraction of a symptomatic tooth. These complications include osteomyelitis, alveolar osteitis, postoperative infection, or septicemia (Jensen et al., 1986; Johri & Piecuch, 2011a; Kunkel et al., 2006; Meurman et al., 1995; Mohammed‐Ali et al., 2010), whereas suggested guidelines for the management of symptomatic and asymptomatic wisdom teeth have remained controversial. Importantly, dentists should concern themselves with whether postoperative pain could be indicative of successful treatment. Dentists also need to determine whether postoperative pain has affected the patient's ability to talk, has resulted in a decrease in the oral intake, or may have caused emotional or psychological discomfort (Jensen et al., 1986; Johri & Piecuch, 2011a; Kunkel et al., 2006; Meurman et al., 1995; Mohammed‐Ali et al., 2010).

The visual analog scale (VAS) score is a common and reliable assessment used for measuring postoperative pain during dental treatments (Katz & Melzack, 1999; Sirintawat et al., 2017). It is presented as a 100−mm horizontal line in which a patient's degree of pain intensity is represented by the left end of the line to indicate “no pain,” while the other end denotes “worst pain imaginable.” On this scale, patients can indicate their level of pain by marking the line (Sirintawat et al., 2017). Practically, the VAS tool is accessible as an online Google document. The tool is particularly user‐friendly because data can be easily collected and conveniently stored. In addition, biomarkers, such as α‐amylase, can be used to predict the severity of pain in conjunction with VAS assessment, which would allow researchers to achieve more reliable and accurate results.

Human parotid saliva is abundant with the α‐amylase (EC 3.2.1.1) which consists of 496 amino‐acid residues, one calcium ion, one chloride ion, and 170 water molecules (Ramasubbu et al., 1996). Notably, this enzyme exerts both main amylolytic activity and bactericidal activity (Bortner et al., 1983). In addition, salivary alpha‐amylase (SAA) provides a potential site for the binding of bacterial adhesions that cause early dental plaque formation (Ramasubbu et al., 1996). In clinical relevance, SAA is a potentially cost‐effective biomarker of psychological stress that can be used to objectively assess the severity of pain for a patient (Christidis et al., 2020; Laurikainen et al., 1988; Nater & Rohleder, 2009; Nederfors & Dahlof, 1996; Nederfors et al., 1994; Speirs et al., 1974; van Stegeren et al., 2006). Regarding oral and periodontal diseases, SAA has been referenced for assessing pain perception and severity in many types of patients (Canigur Bavbek et al., 2021; Castillo‐Felipe et al., 2022; Cotoia et al., 2018; Vahedi et al., 2018; Vaswani et al., 2020; Wittwer et al., 2016; Yennurajalingam et al., 2018). Mechanistically, painful stimuli initially activate symptomatic systems to increase plasma epinephrine or norepinephrine secretions and subsequently increase SAA secretions in response to pain. Saliva can be collected easily and noninvasively; therefore, SAA is believed to be a valuable biomarker in the prediction of pain severity. It can be employed along with the VAS method in dental treatments and operational situations (Ahmadi‐Motamayel et al., 2013; Beaulieu et al., 2010; Furlan et al., 2013; Shirasaki et al., 2007). This study aimed to evaluate and compare the levels of pain intensity and salivary α‐amylase activity in symptomatic and asymptomatic patients after mandibular third molar surgery.

## 2 MATERIALS AND METHODS

### 2.1 Patient preparation and study

A prospective‐cohort collective study was conducted at the Out−Patient Clinic of the Department of Oral and Maxillofacial Surgery, Faculty of Dentistry, Chiang Mai University, Chiang Mai between October 2019 and May 2020. Patients who received mandibular third molar surgery at the clinic agreed to participate in the study and signed a consent form. The inclusion criteria were as follows: patients of an age between 18 and 40 years old, those with a health status categorized as class I by the American Society of Anesthesiologists (Daabiss, 2011), and those presenting a mandibular third molar with a class I position or type A impaction (Pell & Gregory, 1933) with symptoms of acute pericoronitis. The inclusion criteria also identified those who were asymptomatic or who could be treated by simple extraction or surgical removal without bone removal or tooth section. The exclusion criteria eliminated patients with severe acute pericoronitis such as high fever, multiple space involvement, airway obstruction, or a history of salivary gland disease. The exclusion criteria also eliminated those who have used any medication that could affect their salivary glands or who have experienced contraindications for local anesthesia. Those who had used analgesic drugs (such as paracetamol or nonsteroidal anti‐inflammatory drugs), antibiotics, or steroid drugs 1 week before the surgery, or who had experienced difficulties in communicating or writing, were excluded from this study.

The sample size (*n*) was calculated using the method employed in a previous study (Seymour et al., 1985) using the following formula:

$$n=\frac{2\sigma^{2}{\left(Z_{\alpha /2}+Z_{\beta }\right)}^{2}}{{\left(\mu_{t}-\mu_{c}\right)}^{2}},$$

where *Ζ* is a constant, the mean difference (*μ*_1_ – *μ*_2_) of VAS = 3.0, standard deviation (SD, *σ*) = 3.6, confidence level = 95%, power of test = 80, alpha (α) = 0.05, and beta (β) = 0.20. According to the above calculation, the sample size required for the present study was 10 subjects per group. Subsequently, 32 subjects were further divided into two groups: asymptomatic and symptomatic groups (*n* = 16 each). This study was then divided into two parts in which the clinical part involved an assessment of pain level using VAS and the second part involved the collection of saliva specimens for the measurement of SAA activity.

### 2.2 VAS assessment

The subjects in both groups were evaluated in terms of their pain levels using the VAS scoring tool accessible on the Google document online site (https://docs.google.com/forms/d/1QLkQ8mR5eeoxDL1Wk0bWH7FgxSvyb0MFcnkyD6A_cWw/viewform?edit_requested=true/accessed on 22 September 2019) before treatment. Local anesthesia was then administered to the subjects by intramuscular injection of 4% articaine hydrochloride/epinephrine solution (Septacaine® and epinephrine 1:100,000, Septodont). Following that, flushing was achieved with an inferior alveolar nerve block (1.8 ml) and buccal infiltration (0.5 ml). All surgeries were done by the same surgeon who administered either a simple extraction or surgical removal without bone removal or tooth section. The duration of the course of treatment was recorded after initiating the extraction or after the first incision was made. The treatment then lasted until the placement of the last suture but did not exceed 20 min. All subjects were given the same postoperative instructions and received the same type of analgesic paracetamol (500‐mg tablet) when necessary every 6 h, and ibuprofen (400‐mg tablet) when their pain was not relieved after taking the paracetamol. The VAS score was then recorded for both groups after the anesthetic wore off, in the morning at 8.00 am, and before bedtime at 8.00 pm for a period of 1 week after surgery. Notably, when the subjects used analgesic drugs, they were required to record the type, dosage, and duration of time that the drugs were taken.

### 2.3 Collection of saliva and analysis of SAA activity

Subjects in both groups were instructed to neither eat nor drink within 1 h before saliva specimens were collected in order to avoid contamination. Nonstimulated saliva was collected from the floor of the mouth before treatment when the anesthetic wore off and on Days 1 and 7 after surgery, with the use of a sterile plastic pipette. Saliva specimens were spun using a bench‐top fixed‐angle centrifuge (TDZ5−WS, UGAIYA Company) at 3500 rpm, 25°C for 5 min, and the clear saliva supernatant was kept in sterile microcentrifuge tubes (1 ml aliquot) at −20°C until the day of SAA analysis. SAA activity was determined using a coupled enzymatic colorimetric assay kit (α‐Amylase EPS ver.2, Roche Company) that was run according to the manufacturer's operating instructions at the Center for Medical Excellence, Faculty of Medicine, Chiang Mai University (Nater & Rohleder, 2009). In the assay, saliva was diluted with distilled water (1:50) and submitted to an Automated Clin Chem Analyzer (Roche Cobas C501, Roche Company), where 4,6‐ethylidene‐*p*‐nitrophenyl‐α‐d‐maltoheptaosidesubstrate was cleaved under the catalysis of α‐amylase to produce 4,6‐ethylidene‐*p*‐nitrophenol, which will be further cleaved by α‐glucosidase hydrolysis to generate 4,6‐ethylidene and yellow‐colored *p*‐nitrophenol (λ_max_ = 415 nm). The color intensity of the *p*‐nitrophenol formed was directly proportional to the α‐mylase activity.

### 2.4 Statistical analysis

The statistical analyses were conducted using the SPSS program (SPSS version 18.0 for IBM licensed by Chiang Mai University, Thailand). Data are expressed as mean ± standard deviation values (SD) with the significance level indicated by a *p*‐value of < 0.05. Mann–Whitney *U*‐test was used to compare pain levels and SAA activity between groups, for which ^#^ *p* < 0.05 was considered significantly different. Wilcoxon Signed‐Rank test was used to compare levels of pain and SAA activities in subjects of the same group at different times, for which *p < 0.05 was considered significantly different. Spearman test was used to determine any correlation between the VAS‐scored pain level and the SAA activity in both groups.

## 3 RESULTS

### 3.1 Patient information and dental operation

Thirty‐two patients (6 male and 26 female) within the age range of 18–37 years old (24.5 ± 4.7 years) were enrolled in this study and were classified into either the asymptomatic group (*n* = 16, 2 male and 14 female) or the symptomatic group (*n* = 16, 4 male and 12 female) according to their perceptions of pain. Demographic data and the characteristics of the symptomatic and asymptomatic subjects are summarized in Table 1. The subjects in the asymptomatic group were 21.4 ± 4.4 years old with an age range of 18−37 years old and the subjects in the symptomatic group were 25.0 ± 5.1 years old with the age range of years old. Though there were more female patients than male patients, no significant differences were found in terms of ages and numbers for members of the two groups. The operation time periods were similar for both groups and were 8.0 ± 3.5 min in the asymptomatic group and 9.0 ± 2.3 min in the symptomatic group. In the removal procedure of the mandibular third molars, the asymptomatic patients were treated by simple extraction (*n* = 7) and surgery (*n* = 9), while the symptomatic patients were treated by simple extraction (*n* = 8) and surgery (*n* = 8). Interestingly, with the use of VAS assessment, preoperative pain levels in the symptomatic group (5.50 ± 1.87) were significantly higher than those in the asymptomatic group (1.00). However, no significant differences were observed in terms of the postoperative pain levels for members of both groups. In findings, preoperative levels of SAA activity were not different between male and female asymptomatic patients, and they were increased in symptomatic patients whom the male subjects showed higher than the female subjects but not significantly.

**Table 1:** Demographic data and preoperative levels of pain visual analog scale (VAS) and salivary α−amylase (SAA) activity of patients and their treatment for mandibular third molars

Variables	Asymptomatic group	Symptomatic group
Gender (male: female)	2:14	4:12
Age (years): total	24.1 ± 4.4	25.0 ± 5.1
Male	23.6 ± 4.3	23.5 ± 2.5
Female	24.3 ± 4.6	25.5 ± 5.7
Operation time (min)	8.0 ± 3.5	9.0 ± 2.3
Procedure (extraction: surgical removal)	7:9	8:8
VAS of preoperative pain	1.00	5.50 ± 1.87
Preoperative SAA (x10^3^ U/L): total	44.8 ± 27.7	63.1 ± 59.3
Male	44.9 ± 32.3	81.0 ± 66.0
Female	44.7 ± 27.1	57.1 ± 58.8

*Note*: Data are expressed as absolute values or mean ± SD values. Statistical significance was determined using the Mann–Whitney *U*‐test.

### 3.2 VAS assessment of pain intensity

**Figure 1:**
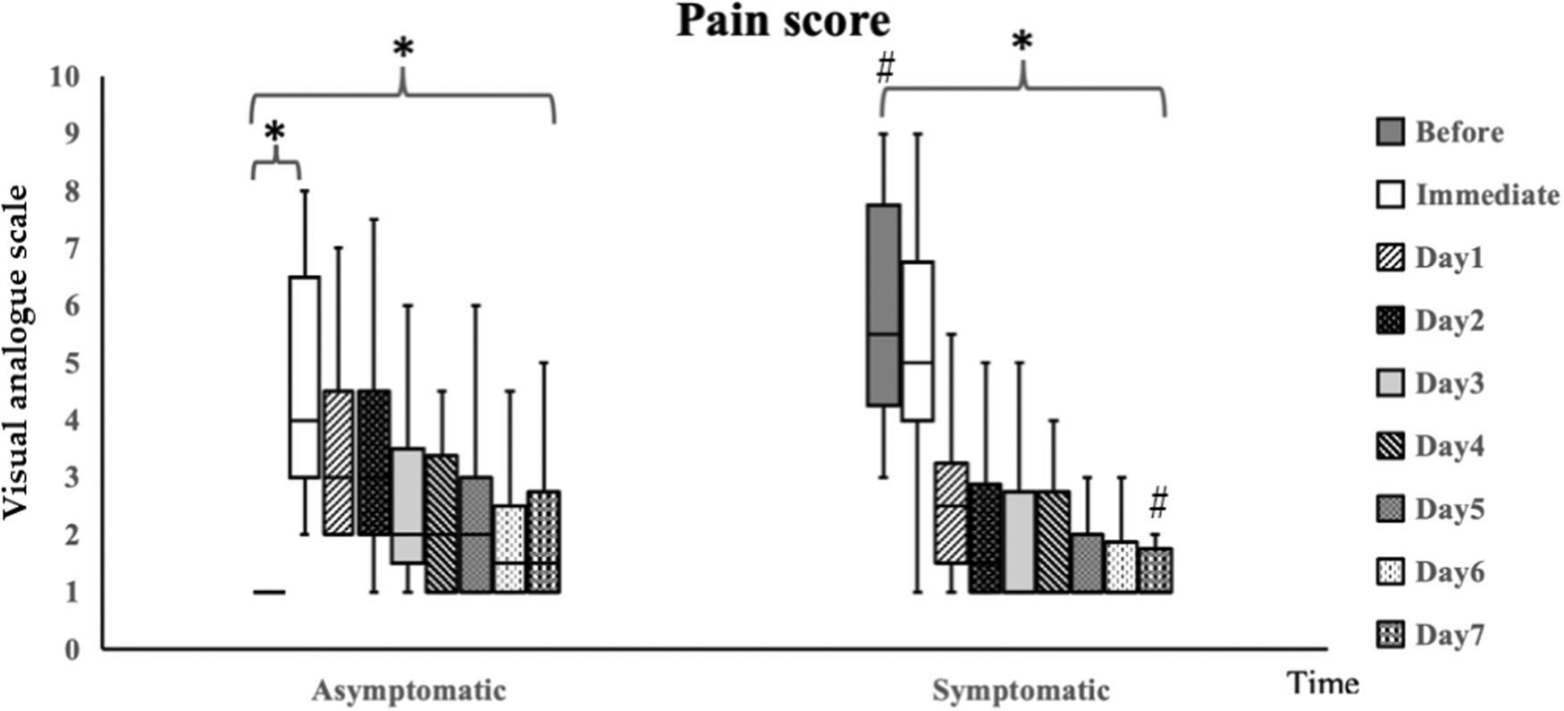
Visual analog scale‐scored pain assessment in asymptomatic and symptomatic patients before and after mandibular third molar extraction. Data are expressed as mean ± SD values (*n* = 16 each). Significant differences were observed when using the Wilcoxon Signed‐Rank test, for which ^#^ *p* < 0.05 when compared to the asymptomatic group and *p < 0.05 when compared to the preoperative pain level.

As is shown in Figure 1, the preoperative pain levels of the symptomatic group were significantly higher than those of the asymptomatic group. Incredibly, the pain levels of the asymptomatic group were immediately increased after tooth extraction when compared to the pretreatment levels (*p* < 0.05). Furthermore, the pain levels of both groups were found to have decreased gradually after the treatment. Accordingly, the members of the symptomatic group did not experience postoperative pain after Day 4 and members of the asymptomatic group did not experience postoperative pain after Day 6. However, on Day 7 of the postoperative period, the level of pain in the symptomatic group was found to have dramatically decreased (*p* < 0.05) when compared to their level of preoperative pain. This value of pain was even lower than it was in the asymptomatic group.

### 3.3 Analysis of SAA activity

**Figure 2:**
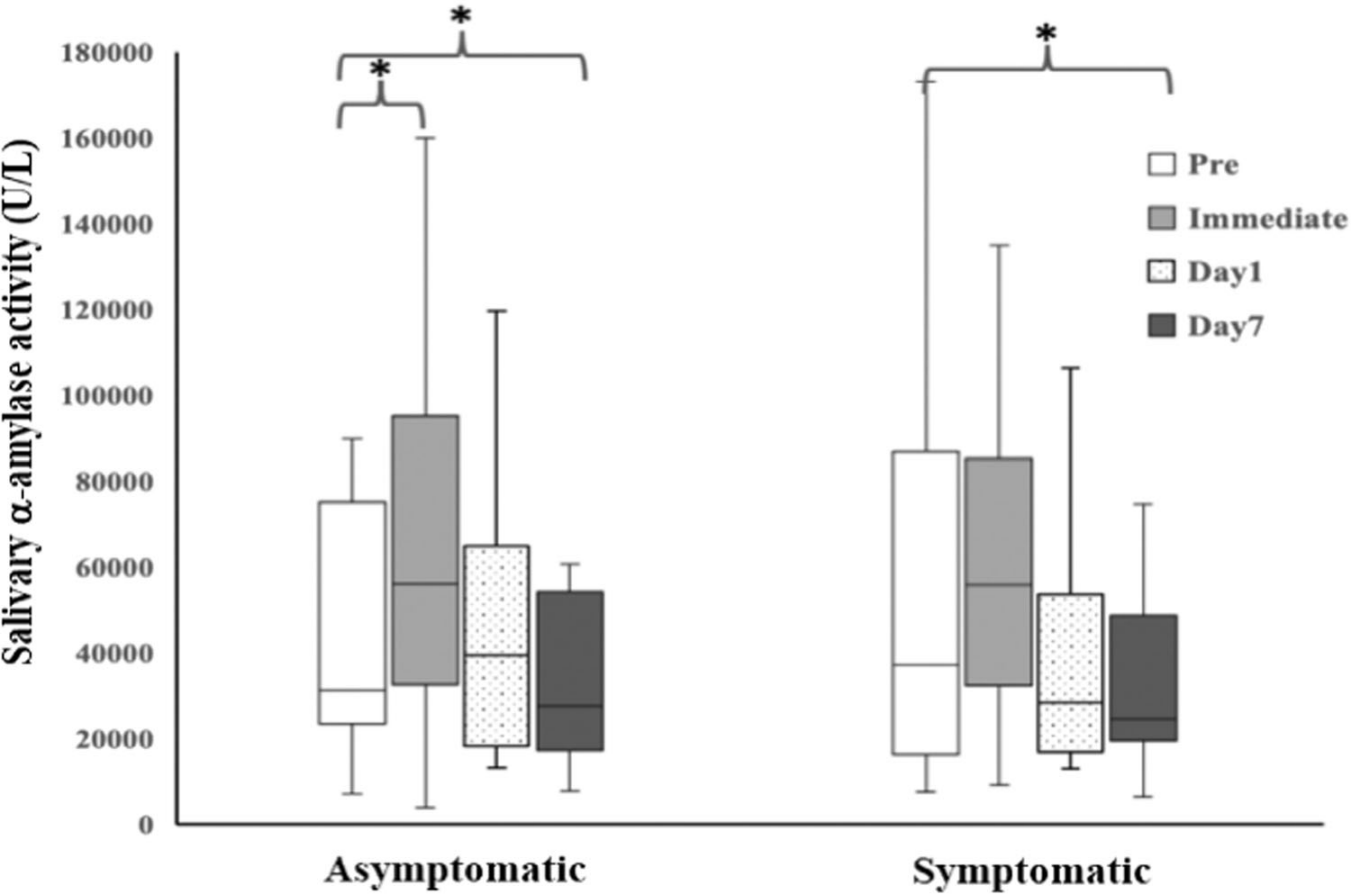
Levels of salivary α‐amylase activities in asymptomatic and symptomatic patients before and after mandibular third molar extraction. Data are expressed as mean ± SD values (*n* = 16 each). Significant differences were observed when the Wilcoxon Signed‐Rank test was employed, for which *p < 0.05 when compared to the preoperative pain level.

The results shown in Figure 2 revealed that the preoperative levels of SAA activities (63.1 ± 59.3 U/L) in the symptomatic group were significantly higher than those (42.3 ± 27.4 U/L) in the asymptomatic group, whereas levels of SSA activities on postoperative Day 7 were not significantly different between these two groups. Unexpectedly, levels of SAA activities on Day 1 of the postoperative period in the asymptomatic group inclined sharply and were significantly higher than the preoperative levels. On the contrary, the levels of SAA activities on Day 1 of the postoperative period were slightly lower than the preoperative levels (*p* > 0.05) and declined gradually until postoperative Day 7. Obviously, the SAA levels on postoperative Day 7 were significantly lower than the preoperative levels for both groups.

**Figure 3:**
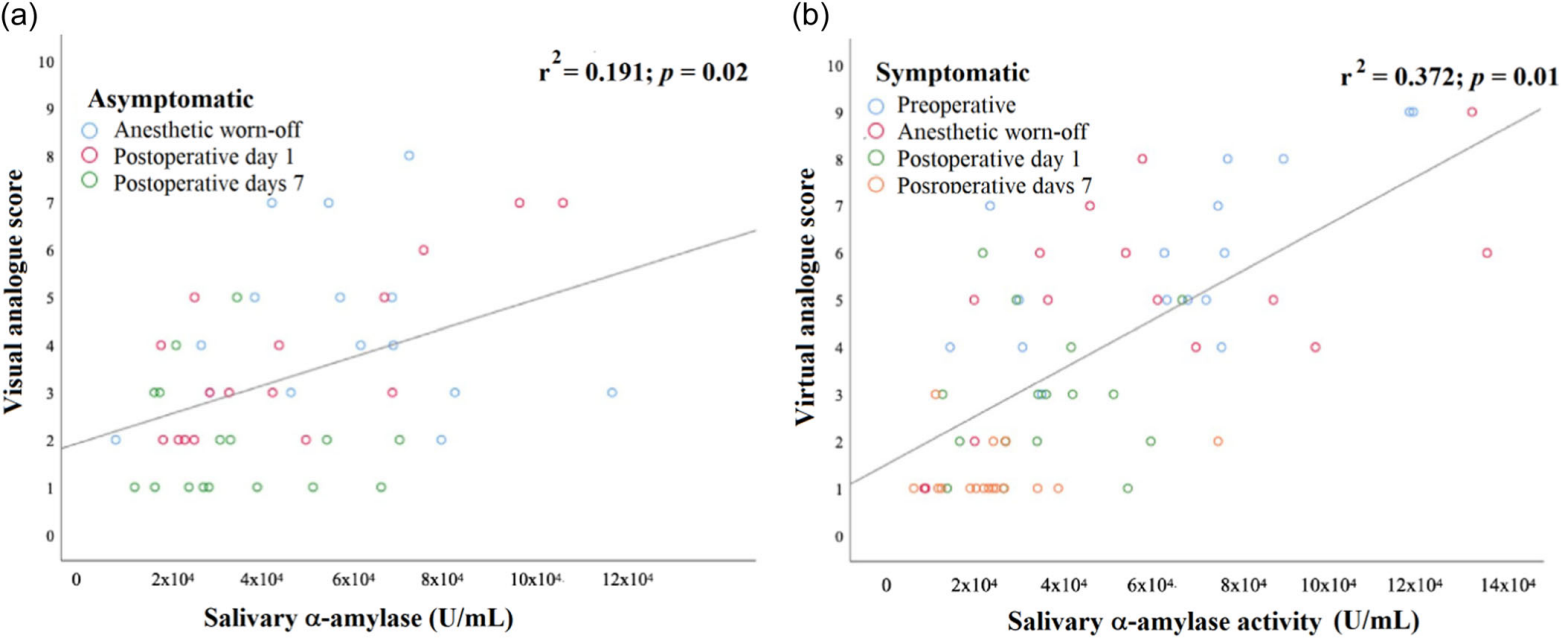
Correlation between pain intensity levels and salivary α‐amylase activity in asymptomatic and symptomatic patients before and after mandibular third molar extraction. Data are presented for asymptomatic (a) and symptomatic (b) groups. Significant difference was observed when using the Spearman test, for which *p* < 0.05 was considered significantly different.

In addition, Spearman correlation analysis revealed a positive correlation between pain levels and the levels of SAA activities for members of each group and among both groups of asymptomatic and symptomatic patients before and after mandibular third molar extraction (Figure 3).

Analgesic drugs (in terms of tablets per day) taken by asymptomatic and symptomatic patients for all periods are shown in Table 2. The data reveal that both of the patient groups took the largest doses of the drugs on postoperative Day 1, while the dosage was reduced according to the time and any differences in the number of tablets taken for members of both groups.

**Table 2:** Amounts of analgesic drugs (mean ± SD) taken by asymptomatic and symptomatic patients on each postoperative day for 7 days

	Analgesic drug (tablets)							
Group	**0**	**1**	**2**	**3**	**4**	**5**	**6**	**7**
Asymptomatic	0.67 ± 0.76	0.72 ± 1.02	0.56 ± 0.91	0.34 ± 0.60	0.31 ± 0.64	0.12 ± 0.33	0.29 ± 0.21	0.03 ± 0.03
Symptomatic	0.67 ± 0.73	0.50 + 0.91	0.40 + 0.79	0.21 ± 0.55	0.18 ± 0.59	0.12 ± 0.33	0.06 ± 0.24	0.03 ± 0.17

**Figure 4:**
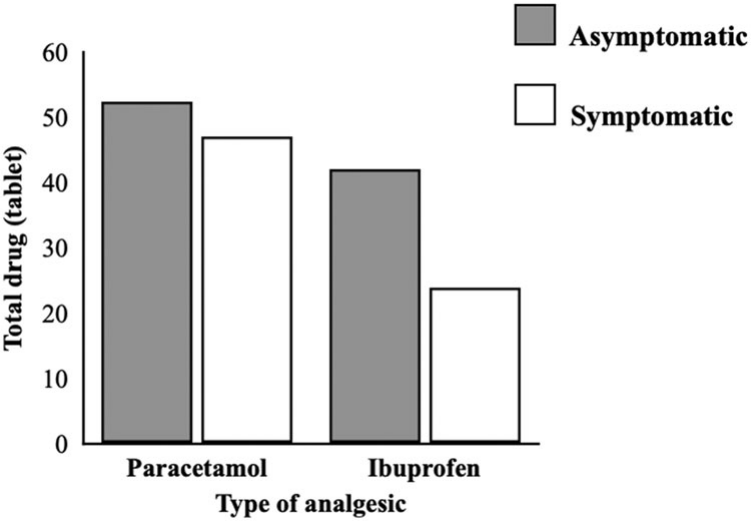
Average values in terms of the total dosage of analgesic drugs (paracetamol and ibuprofen) taken by asymptomatic and symptomatic patients on each postoperative day for 7 days.

Moreover, a statistically significant correlation was observed between the levels of pain and the number of analgesic tablets taken (*p* < 0.05), for which the dosage of the analgesic drug increased along with the severity of pain (*r* = 0.475, *p* < 0.001) (Figure 4).

### 3.4 Evaluation of complications

The complication rate occurred in a total of seven patients (three in the asymptomatic group and four in the symptomatic group) who felt uncomfortable after the treatment. Subjects claimed to experience postoperative pain, a limited ability to open their mouths, and difficulties in eating or swallowing (Table 3); however, they recovered without treatment within a period of 1−2 weeks. In addition, the worst complication was identified as a postoperative infection that was found in one asymptomatic patient. This was resolved after an incision was made and the wound was allowed to drain. Subsequently, the subject was administered antibiotic treatment over a period of 1 week.

**Table 3:** Postoperative complications in asymptomatic and symptomatic patients (*n* = 16 each) before and after mandibular third molar extraction

Complications	Asymptomatic group	Symptomatic group
Uncomfortable	3 of 16	4 or 16
Postoperative infection	1 of 16	None
Total	4 of 16	4 of 16

## 4 DISCUSSION

The controversial topic in the field of oral maxillofacial surgery is whether or not to extract teeth immediately in cases of acute infection or for symptomatic patients. Previous studies have shown that the extraction of the presence of acute infection may induce the bacteria to seed into the fascia space, potentially resulting in a life‐threatening infection (Johri & Piecuch, 2011b) and a higher degree of postoperative pain than among the asymptomatic patients (Seymour et al., 1985). However, some studies have supported immediate extraction of the tooth in cases of acute infection in order to remove the source of the infection, relieve the patient's pain and prevent any severe complications such as deep fascia space infection or compromised airways (Fasalulla et al., 2017; Rud, 1970; Wainwright, 1940).

In the present study, we have determined that there was no difference in the levels of pain assessed by the VAS between symptomatic and asymptomatic patients following the surgical removal of the mandibular third molar. This finding was similar to that of the study conducted by Hall and colleagues which reported that an immediate extraction of the teeth would achieve a greater reduction in the level of pain and the amount of swelling experienced than as a consequence of the supportive treatment (Hall et al., 1968). Moreover, Fasallula et al. reported that the immediate treatment was more likely to reduce the level of pain of the subject, the duration of treatment, the number of days a patient was admitted to the hospital, the number of analgesic drugs required, and any incidence of postoperative incisions that were accompanied by drainage (Fasalulla et al., 2017). However, members of the pericoronitis group were found to have experienced higher postoperative pain levels than members of the asymptomatic group. Consequently, it is believed that some residual infections may elicit prostaglandin synthesis and cause prior sensitization of free nerve endings that would induce greater levels of pain than was experienced by members of the symptomatic group (Seymour et al., 1985). With regard to any potential limitations of this study, the duration of the treatment and the depth of the teeth were not controlled and may have led to inaccurate results. Herein, we have controlled the duration of the treatment in each case to not be longer than 20 min in order to produce more accurate results.

In addition, the findings of our study have shown that the duration of pain after tooth extraction was highest after the anesthetic wore off and would gradually decrease until it returned to normal levels on Week 1, as has been reported in previous studies (Fasalulla et al., 2017; Seymour et al., 1985). The pain levels of the asymptomatic group returned to normal levels on postoperative Day 6, while those of the symptomatic group returned to normal levels on postoperative Day 4. As a result, members of the symptomatic group were more likely to see their pain relieved than those of the asymptomatic group. In addition, on postoperative Day 7, the symptomatic group had 25% of its patients reporting pain, while the asymptomatic group reported that the number of patients reporting pain remained at 50%, as is shown in Figure 4. Therefore, the findings of our study confirmed that the immediate mandibular third molar extraction procedure could eliminate the cause of the infection, help patients feel more comfortable, and relieve pain, while not recording any differences in the postoperative pain experienced by symptomatic and asymptomatic patients. SAA is produced by the salivary glands and activated by increasing plasma norepinephrine levels during periods of psychological stress (Campos et al., 2011). Pain is recognized as a stressful agent that induces the activation of epinephrine and norepinephrine (Shirasaki et al., 2007), leading to an increase in SAA secretions (Campos et al., 2011). With regard to the findings of previous studies, the levels of SAA were significantly correlated with the degree of pain severity assessed by VAS (Ahmadi‐Motamayel et al., 2013; Anggraeni et al., 2017; Gutierrez Corrales et al., 2017; Shirasaki et al., 2007). Interestingly, SAA activity might decrease in relation to age in subjects over 40 years old, which may have affected the results of that study; therefore, we chose to include subjects aged between 18 and 40 years old. Herein, SAA was selected as a biomarker and predictor of pain levels and was found to be significantly correlated with levels of pain intensity, which was consistent with the findings of previous studies. During the preoperation period for the extraction of the mandibular third molar, no pain was reported in the asymptomatic group and initial levels of SAA activities were determined to be normal. Afterward, the tooth extraction procedure caused pain, which then induced the SAA activity levels to rise up and gradually decrease over time and after the pain subsided. In contrast, the pain levels of the symptomatic patient group were already high at the pretreatment period, while the levels of SAA activity were also found to be high. After extraction of the infected mandibular third molar tooth was removed as the source of the pain, pain levels gradually decreased along with a decrease in SAA activity levels. Until Day 7 of the postoperative period, the levels of SAA activities among members of both groups returned to a normal range (Nater & Rohleder, 2009). As a recommendation, SAA can be considered a potential biomarker that can be used to assess pain levels. This is because it is considered reliable and can be easily and noninvasively collected.

Importantly, the pain levels evaluated in this study were significantly correlated with the amount of medication used, which was similar to the findings of previous studies (Fasalulla et al., 2017; Seymour et al., 1985). Furthermore, the amount of analgesic drugs taken can be a key factor that can be used to predict the severity of a patient's pain. Two separate studies conducted by Fasalula et al. (2017) and Seymour et al. (1985) have revealed that the symptomatic and asymptomatic groups recorded taking the greatest amounts of drugs on Day 1 of the postoperative period, while the number of drugs taken then decreased and the pain levels subsequently decreased. Moreover, another previous study (Fasalulla et al., 2017) reported that the immediate treatment in the symptomatic group lessened the dose and duration of the drugs taken as opposed to the administration of the supportive treatment. Herein, we found that the amounts of analgesic drugs taken by the two patient groups were not different, but some significant observations were made regarding the type of drugs used. The paracetamol doses used among both groups were equal but the symptomatic group took less ibuprofen than the asymptomatic group. It can be assumed that after treatment, the symptomatic group experienced a greater reduction in pain; therefore, a lower dose of ibuprofen was administered. Thus, the immediate extraction of a problematic tooth during periods of acute pain would not only eliminate the cause of the infection but also reduce the number of analgesic drugs required.

Another concern is the risk of postoperative complications after the extraction procedure, during acute periods of pain, or among symptomatic patients (Johri & Piecuch, 2011b). Anyanechi et al. reported that the surgical removal of the mandibular third molar in symptomatic and asymptomatic patients was associated with more postoperative complications than in the symptomatic group (Anyanechi & Saheeb, 2017). The occurrence of more complications may be due to some residual infections in the soft tissue and bones around the teeth, which could increase incidences of postoperative infection and induce greater inflammatory responses (Anyanechi & Saheeb, 2017; Seymour et al., 1985). Likewise, Anyanechi et al. found that lower socioeconomic groups and the duration of treatment are significantly statistically correlated with postoperative complications (Anyanechi & Saheeb, 2017). Nonetheless, several reports have supported immediate treatment for acute pain because leaving an infected tooth in place may lead to more serious complications. These complications can include deep space infections that lead to airway obstructions and an increased risk of septicemia. These serious complications are difficult to treat and can be considered life‐threatening conditions. The present study has identified the presence of mild and severe complications among members of both groups of patients following mandibular third molar extractions. For instance, a mild complication would include the degree of discomfort experienced by the patient, dysphagia, increased pain, and decreased ingestion, all of which can be resolved without treatment in 1–2 weeks. Incidences of severe complications include one symptomatic patient who experienced a postoperative infection and who was treated by incision and drainage combined with antibiotic administration. After 1 week of this treatment, the patient improved by way of decreases in both pain and swelling. With regard to postoperative complications, the decision of whether to treat members of the symptomatic group would be made if severe postoperative complications occurred and when the dentist had to carefully manage this condition.

The results from this study may be used to consider guidelines for treating patients with acute pain. Immediate tooth extraction will also help eliminate the source of infection, reduce the pain experienced by the patient, and reduce the doses of drugs taken. However, some patients have indicated a relative inclination to delay tooth extraction for reasons of uncontrolled systemic disease, taking anticoagulant drugs, being an immune‐compromised patient, or the perceived inefficiency of a local anesthetic (Johri & Piecuch, 2011b). Consequently, supportive forms of treatment included the taking of analgesic or antibiotic drugs. Nonetheless, the source of the infection should be eliminated as soon as possible so as to avoid any complications. Importantly, anti‐hypertensive drugs, asthma medication, and any medications that contain adrenergic agonists or antagonists can strongly impact SAA levels; therefore, patients who took these drugs were excluded from the study. Notably, analgesic drugs (e.g., paracetamol) and nonsteroidal anti‐inflammatory drugs (e.g., aspirin, ibuprofen, and indomethacin) do not influence SAA levels (Rohleder & Nater, 2009). Herein, there were more women than men, 2:14 in the asymptomatic group and 4:12 in the symptomatic group, who were willing to take part in this study. Alpha‐amylase is defined as a biocatalyst and biomarker of pancreatic function, the sympathetic nervous system, and pain severity, which can be quantified in terms of absolute activity (unit/liter) and specific activity (unit/mg protein). The amount of SAA is mostly expressed as unit/liter as has been mentioned in previously published research work (Ahmadi‐Motamayel et al., 2013; Rohleder & Nater, 2009; Shirasaki et al., 2007). However, factors that may influence SAA include gender, age, smoking, personal fitness, personality chronic stress, and psychopathology (Rohleder & Nater, 2009). In addition, the female menstrual cycle may affect levels of SAA according to the induction of secretory catecholamines (Rohleder & Nater, 2009).

Based on the findings of this study, the decision to administer treatment in symptomatic patients must be based on several key factors. The first factor would be dependent upon the patient such as whether they have experienced a systemic condition, the type of drug they may be taking, their cooperative attitude after treatment, and the socioeconomic conditions of the patient. Importantly, each of the above‐mentioned factors should be considered before treatment is initiated. The next factor involves an assessment of the severity of infection in order to determine the urgency of treatment and the patient's oral hygiene habits. These are a secondary set of factors that may contribute to the success of treatment. The last factor focuses on the dentist or surgeon who should display the ability and experience to manage the patients' experience including their readiness to deal with complications that may arise after treatment. The results from this study may also be used as evidence‐based findings that could assist medical professionals in determining whether and how symptomatic patients should be treated. This would be done so as to achieve the greatest benefits for both the patients and the dentists. In terms of the potential limitations of our study, a short period of follow‐up may not have allowed researchers to consider any postoperative complications after a period of 1 week. Furthermore, the inclusion of wisdom teeth in the upright position, or the class I position A, may have limited the positions of some of the other teeth. Additionally, SAA activity can easily be changed according to the emotional condition or level of stress experienced by the patient, which may have then led to inaccurate results.

In conclusion, no differences were observed in postoperative pain levels between symptomatic and asymptomatic patients following mandibular third molar treatments. Thus, it is suggested that the infected teeth be removed as soon and as safely as possible in order to address the acute levels of pain experienced by the subjects. Early extraction of infected teeth hastens the recovery process, promotes pain relief, eliminates the source of the infection, and prevents the occurrence of further severe complications. Specifically, salivary alpha‐amylase is recommended as a biomarker that can be used to assess pain severity.

## AUTHOR CONTRIBUTIONS

**Wanvipa Surin**: Conceptualization; clinical investigation; collection of saliva specimens; and writing original draft‐manuscript. **Piyanart Chatiketu**: Conceptualization and statistical analysis. **Nuntouchaporn Hutachok**: Methodology and α‐amylase analysis. **Somdet Srichairatanakool**: Conceptualization; methodology and discussion. **Vuttinun Chatupos**: Conceptualization; supervision; visualization and correspondence to reviewers and the editor of the journal.

## CONFLICT OF INTEREST

The authors declare no conflict of interest.

## ETHICS STATEMENT

The experiments were undertaken with the understanding and written consent of each subject and according to the ethical principles, including the World Medical Association Declaration of Helsinki (version 2002) and the additional requirements, if any, of the country where the research has been carried out. The study has been independently reviewed and approved of by the Local Human Experimentation Committee of the Faculty of Dentistry, Chiang Mai University (Study Code No. 39/2018).

## Data Availability

Data sharing is not applicable to this article as no new data were created or analyzed in this study.
